# Knowledge translation strategies used for sustainability of an evidence-based intervention in child health: a multimethod qualitative study

**DOI:** 10.1186/s12912-024-01777-4

**Published:** 2024-02-17

**Authors:** Christine E. Cassidy, Rachel Flynn, Alyson Campbell, Lauren Dobson, Jodi Langley, Deborah McNeil, Ella Milne, Pilar Zanoni, Megan Churchill, Karen M. Benzies

**Affiliations:** 1 School of Nursing, Faculty of Health, Dalhousie University, 5869 University Avenue, B3H 4R2 Halifax, NS PO Box 15000, Canada; 2 School of Nursing and Midwifery, Brookfield Health Sciences Complex, University College of Cork, College Road, T12 AK54 Cork, Ireland; 3 Faculty of Nursing, University of Prince Edward Island, 550 University Avenue, HSB Room 116, C1A 4P3 Charlottetown, PE Canada; 4 Faculty of Nursing, Edmonton Clinic Health Academy, University of Alberta, Level 3, 11405 87 Avenue, T6G 1C9 Edmonton, AB Canada; 5 Faculty of Health, Dalhousie University, 5790 University Avenue, B3H 1V7 Halifax, NS Canada; 6 Faculty of Nursing , University of Calgary, 2500 University Drive NW, T2N 1N4 Calgary, AB Canada; 7 Strategic Clinical Networks, Alberta Health Services, 10101 Southport Road SW, T2W 3N2 Calgary, AB Canada; 8 Faculty of Nursing, Departments of Pediatrics and Community Health Science, Cumming School of Medicine, University of Calgary, 2500 University Drive NW, T2N 1N4 Calgary, AB Canada; 9 Department of Pediatrics, IWK Health, 5980 University Ave #5850, B3K 6R8 Halifax, NS Canada

**Keywords:** Sustainability, Implementation science, Knowledge translation, Family Integrated Care, Pediatrics

## Abstract

**Background:**

Sustainability of evidence-based interventions (EBIs) is suboptimal in healthcare. Evidence on how knowledge translation (KT) strategies are used for the sustainability of EBIs in practice is lacking. This study examined what and how KT strategies were used to facilitate the sustainability of Alberta Family Integrated Care (FICare)™, a psychoeducational model of care scaled and spread across 14 neonatal intensive care units, in Alberta, Canada.

**Methods:**

First, we conducted an environmental scan of relevant documents to determine the use of KT strategies to support the sustainability of Alberta FICare™. Second, we conducted semi-structured interviews with decision makers and operational leaders to explore what and how KT strategies were used for the sustainability of Alberta FICare™, as well as barriers and facilitators to using the KT strategies for sustainability. We used the Expert Recommendations for Implementation Change (ERIC) taxonomy to code the strategies. Lastly, we facilitated consultation meetings with the Alberta FICare™ leads to share and gain insights and clarification on our findings.

**Results:**

We identified nine KT strategies to facilitate the sustainability of Alberta FICare™: Conduct ongoing training; Identify and prepare local champions; Research co-production; Remind clinicians; Audit and provide feedback; Change record systems; Promote adaptability; Access new funding; and Involve patients/consumers and family members. A significant barrier to the sustainability of Alberta FICare™ was a lack of clarity on who was responsible for the ongoing maintenance of the intervention. A key facilitator to sustainability of Alberta FICare was its alignment with the Maternal, Newborn, Child & Youth Strategic Clinical Network (MNCY SCN) priorities. Co-production between researchers and health system partners in the design, implementation, and scale and spread of Alberta FICare™ was critical to sustainability.

**Conclusion:**

This research highlights the importance of clearly articulating who is responsible for continued championing for the sustainability of EBIs. Additionally, our research demonstrates that the adaptation of interventions must be considered from the onset of implementation so interventions can be tailored to align with contextual barriers for sustainability. Clear guidance is needed to continually support researchers and health system leaders in co-producing strategies that facilitate the long-term sustainability of effective EBIs in practice.

**Supplementary Information:**

The online version contains supplementary material available at 10.1186/s12912-024-01777-4.

## Background

Given that the nursing profession represents the largest percentage of the healthcare workforce, nurses have considerable potential to translate evidence into practice and improve patient and health system outcomes [1, 2]. Evidence-based interventions (EBIs; e.g., clinical practice guidelines, clinical pathways, innovations, models of care) are useful for translating evidence into nursing practice; however, the availability of EBIs does not guarantee that they will be successfully implemented, adopted, and sustained in practice [3, 4]. The field of implementation science has a robust literature on knowledge translation (KT) strategies to promote the implementation of EBIs into practice [5]. KT strategies are defined as “methods or techniques used to enhance the adoption, implementation, and sustainability of a clinical program or practice” [6]. Examples of KT strategies include educational approaches, audit and feedback, and clinical champions [7]. There is an abundance of evidence on the use of KT strategies [8–11] for the implementation of various EBIs with different stakeholders (e.g., nurses, physiotherapists, physicians) [12, 13], across different health contexts [14, 15]. To date, this literature focuses primarily on the use of KT strategies for the implementation process of EBIs into different healthcare contexts. There is limited consolidated empirical evidence on *what* and *how* KT strategies are used for the sustainability of EBIs in healthcare institutional settings (e.g., hospitals, long-term care organizations).

Sustainability is conceptualized as both a process and implementation outcome and is a priority issue for health services research [16, 17]. Moore et al. describe sustainability as after a defined period of time, the program, clinical intervention, and/or implementation strategies continue to be delivered and/or individual behavior change (i.e., clinician, patient) is maintained; the program and individual behavior change may evolve or adapt while continuing to produce benefits for individuals/systems. The sustainability concept differs from scale up and spread, which Greenhalgh and Papoutsi [18] define as building infrastructure to support full scale implementation (scale up), and replication of an intervention (spread). Sustainability of EBIs continues to be suboptimal across healthcare institutions, due to the lack of understanding of strategies available to support sustainability [19]. Our recent scoping review synthesized 25 studies and found that training, education, and the development of interrelationships between researchers and knowledge users are the most common types of KT strategies used to sustain EBIs [20]. A key finding from our review was the need for clearer description and reporting of KT strategies used for the sustainability of EBIs and research that describes *how* to use KT strategies to sustain EBIs [20]. This information is critical to support nurses and nurse leaders to implement and sustain EBIs in a variety of healthcare contexts.

To address the knowledge gaps found in our scoping review, this current study aimed to explore what and how KT strategies are used to facilitate the sustainability of one EBI that has been scaled and spread across the context of Alberta Health Services (AHS), Canada. Given its robust evidence-base and successful implementation across the province of Alberta, Canada, we selected Alberta Family Integrated Care (FICare)™ as the case EBI for this study. Alberta FICare™ is a theoretically driven, psychoeducational model of care that enhances family-centered care practice, driven by the multi-disciplinary team (largely comprised of nurses), and empowers parents of infants admitted to the neonatal intensive care unit (NICU) with knowledge, skills, and confidence to facilitate an earlier discharge home [21, 22]. Modeled off a program in Estonia, a model of FICare for level 3 NICUs was first implemented as a pilot study in 2011 at Mount Sinai Hospital in Toronto, ON. Alberta FICare™ was adapted from the level 3 NICU model and subsequently implemented and evaluated in 10 level 2 NICUs across Alberta in a cluster randomized controlled trial (cRCT) [23–25]. Successful implementation of Alberta FICare™ was shown to decrease length of stay (LOS) by 2.55 days without significant increases in readmissions and emergency department (ED) visits compared to moderate to late preterm infants in a standard care group [23]. Parents who engaged with Alberta FICare™ reported reduced psychological distress and improved confidence in caring for their infant [26, 27]. This increased confidence and positive experience gained from the integration of Alberta FICare™ into practice has the potential to improve infant-parent relationships, which ultimately supports communication skill development in infants [21], improved neurodevelopment in preterm infants [28], and increased confidence in parents’ transition home with their infant [26, 27]. In 2019, Alberta FICare™ spread and scale was initiated for all 14 NICUs across the province [29]. Previous research has been conducted to explore barriers and facilitators to implementation of the Alberta FICare™ in clinical practice [22]; however, no research has been conducted to examine *what* and *how* KT strategies were used to facilitate the sustainability of Alberta FICare™ across the province.

### Research purpose

This study examined what and how KT strategies were used to facilitate the sustainability of Alberta FICare™ in level II and level III NICUs across Alberta, Canada.

Our research objectives were to:

1. Identify *what* and *how* KT strategies are used to support the sustainability of Alberta FICare™; and.
2. Understand the perceived barriers and facilitators to using KT strategies for the sustainability of Alberta FICare™.

## Methods

We conducted a multimethod qualitative study across three sequential phases: (1) environmental scan of relevant documents (policies, guidelines, meeting notes, protocols, etc.) on the use of KT strategies to support the sustainability of Alberta FICare™; (2) key informant interviews with nurses, decision makers, administrators, and operational leaders with experience implementing and sustaining Alberta FICare™; and (3) consultation with the Alberta FICare™ leads to share and gain insights and clarification on our findings. We defined sustainability as use of the EBI beyond 1 year of initial implementation of Alberta FICare™ at the specific site [30]. Alberta FICare™ was initially implemented in five test sites involved in the cRCT. From there, the EBI was spread and scaled to all control sites involved in the cRCT and remaining NICUs in the province, for a total of 14 NICUs. The researchers responsible for data collection and analysis (CEC, RF, LD, EM, JL) were external to the Alberta Health Services NICU setting and did not have any relationships with participants.

### Phase 1: environmental scan

An environmental scan is a passive strategy for externally examining a phenomenon of interest using existing sources of information [31]. Our environmental scan included a systematic approach to searching relevant documents, extracting data, and synthesizing the findings.

#### Search strategy

We sourced a range of documents for the environmental scan, including project management plans, open-access journal articles, knowledge user presentations, and meeting documents on initial implementation from the cRCT and scale and spread provided by the Alberta FICare™ Project Team. Further, we explored the AHS website on Alberta FICare™ to identify items related to sustainability. We held two meetings with the Alberta FICare™ Project Team to identify any additional documentation for the environmental scan. During these meetings, it was agreed that any documents that had any personal identification (i.e., names of individuals) would be excluded or de-identified for analysis.

#### Data extraction

We created a data extraction form in Excel to collect relevant information related to: document source type (protocol, policy, meeting notes, etc.); Authors; Year; Definition of sustainability concept or phase (if reported); Type of KT strategy used according to the Expert Recommendations for Implementing Change (ERIC) taxonomy [7]; KT strategy description using the Aims, Ingredients, Mechanism, Deliver (AIMD) Framework [32]; Adaptations/modifications of KT strategy from implementation to sustainability; Reported barriers and facilitators to sustainability; Reported Outcomes. One reviewer (JL) extracted all data using the data extraction form. Collectively, the research team met and reviewed the extracted data to determine any additional information that needed to be extracted.

#### Data analysis

We produced descriptive numerical summaries of the quantitative data (i.e., frequency of document types, KT strategy, barriers and facilitators, outcomes, etc.). Next, two team members (CC, JL) conducted deductive content analysis to categorize the KT Strategies using the ERIC taxonomy consisting of 73 strategies [7, 33]. If data did not map onto the ERIC taxonomy, we coded it under “other”. Findings are reported narratively and in tabular formats.

### Phase 2: key informant interviews

The environmental scan was complemented by key informant interviews using a qualitative descriptive design [34]. The objective of the key informant interviews was to explore KT strategies used to facilitate the sustainability of Alberta FICare™ from the perspectives of nurses, decision makers, administrators, and operational leaders. Ethics approval was granted by the University of Alberta Health Research Ethics Board (CHREB #Pro00116834) and the Covenant Health Research Centre.

#### Participants

To meet the inclusion criteria of a key informant, participants had to have experience with implementing and sustaining Alberta FICare™. Informants were contacted via email by the Executive Director of the Maternal Newborn Child & Youth (MNCY) Strategic Clinical Network (SCN)™, and a follow up email approximately two and four weeks after if a response was not received. Interested participants contacted the Research Assistant (RA) who arranged an online interview via Zoom.

#### Procedure

We developed a semi-structured interview guide based on the Consolidated Framework for Sustainability (CFS) [35] to explore barriers and facilitators to KT strategy use for the sustainability of Alberta FICare™ (Objective 2; See Appendix 1 for Interview Guide). We included prompts of specific ERIC Taxonomy strategies based on findings from our scoping review of KT strategies used to sustain EBIs. Open-ended questions were also included to explore additional strategies that may not be included in the ERIC Taxonomy. Further, additional questions were posed to seek clarification or additional information based on findings from the environmental scan. The interviews were conducted by two researchers (an RA and principal or co-investigator). Interviews lasted between 45 and 60 min. Participants provided written, informed consent before the interview.

#### Data management and analysis

Audio-recordings for all interviews were transcribed verbatim and de-identified. Data were managed and analyzed using NVivo 12 [36]. First, two members of the research team (LD, EM) conducted deductive content analysis [33] to code similar statements related to KT strategies used for sustaining Alberta FICare™. Strategies were deduced according to the ERIC taxonomy of implementation strategies, which consists of 73 distinct strategies categorized into 9 separate clusters [7]. If data did not map onto the ERIC taxonomy, we coded it under “other”. Next, we used the CFS to code similar statements related to the barriers and facilitators to using the KT strategies [35]. Two research team members (LD, EM) cross-referenced their analyses and compared their preliminary findings. Together, they developed a final set of themes and summaries which were reviewed and refined by two other research team members (CC, RF). Any discrepancies were resolved through team discussion with co-leads (CC, RF).

### Phase 3: consultation with alberta FICare™ leaders

To enhance the methodological rigour of our environmental scan and key informant interviews, we consulted with key Alberta FICare™ Leads to share our findings, gain insights, and seek clarification. Specifically, we worked closely with the Principal Investigator/Scientific Lead of Alberta FICare™ (KB), the Scientific Director of the AHS MNCY SCN (DM), and Project Manager of Alberta FICare™ (PZ). This consultation involved two virtual meetings to discuss relevant documents for the environmental scan and clarify key findings. The Alberta FICare™ Leads also provided insights on our key findings via written feedback and are co-authors on this paper.

## Results

### Phase 1: environmental scan

The environmental scan identified three ERIC taxonomy KT strategies [7] used to facilitate EBI (Alberta FICare™) sustainability: (1) conduct ongoing training; (2) identify and prepare champions; and (3) research co-production with the MNCY SCN (Table 1). The training and education strategies targeted all clinicians and unit clerks in the NICU with the goal of increasing the level of knowledge on Alberta FICare™. These strategies included 1–2 h of in-person training for sites involved in the cRCT and transitioned to asynchronous online education modules for sites involved in the scale and spread. The second strategy included managers identifying clinical champions within their care setting to take on a larger role and act as facilitators to support sustainability of Alberta FICare™ at the point-of-care with their nurse colleagues. Clinical champions received 3–4 h of educational training on Alberta FICare™. Lastly, the provincial scale and spread of Alberta FICare™ was completed using a co-production approach with the MNCY SCN to support the sustainability of FICare. This included quarterly fidelity audits and debriefs with Local Site Implementation Teams where co-leads and the project manager answered questions and recommended strategies to strengthen implementation.

### Phase 2: key informant interviews

#### Characteristics of participants

Of the five individuals interviewed, four identified themselves as female. The five interviewees held diverse roles in both AHS and Covenant Health. Covenant Health is contracted by AHS to deliver healthcare services and is part of Alberta’s integrated health system [37]. We interviewed two program managers, one unit manager, one clinical nurse educator, and one clinical project manager. Three participants spoke from the Edmonton Zone and two from the Calgary Zone. Three participants worked in a level 2 NICU and two worked in a level 3 NICU.

**Table 1:** Demographic information of participants

Gender		Role		Time in Role		Level of NICU		Zone of NICU	
Female	*n* = 4	Program Manager	*n* = 2	< 1 year	*n* = 3	Level 2	*n* = 3	Edmonton	*n* = 3
Male	*n* = 1	Unit Manager	*n* = 1	2–4 years	*n* = 2	Level 3	*n* = 2	Calgary	*n* = 2
		Clinical Nurse Educator	*n* = 1						
		Clinical Program Manager	*n* = 1						

#### Types of KT strategies used for sustainability

From the key informant interviews, all data mapped onto the ERIC Taxonomy. We identified a total of eight distinct ERIC taxonomy KT strategies used to support the sustainability of Alberta FICare™ (Table 2). Three KT strategies were the same strategies identified in the environmental scan (conduct ongoing training, identify and prepare champions, research co-production). The types of KT strategies used varied by site; however, staff training was the only strategy reported in all interviews. Following staff training, the two most reported KT strategies were audit and feedback and optimizing record systems to support integration of Alberta FICare™ into the workflow. The eight KT strategies, categorized into the ERIC taxonomy, reported for the sustainability of Alberta FICare™ are:

**Table 2:** KT strategies used for the sustainability of alberta FICare™ and reported barriers and facilitators to their use

ERIC Taxonomy KT Strategy	Description of KT Strategy	Barriers (B) and Facilitators (F) to use of KT Strategy	CFS Construct^28^
***Identified in Environmental Scan and Key Informant Interviews***			
Conduct ongoing training	• Online education modules for nurses • Asynchronous • Webpage	• Increase in workload to complete training (B) • Lack of accessibility to ongoing education materials (B)	*Negotiating initiative processes*
Identify and prepare champions	• Identification of nursing staff within each care setting to take on a champion role to help sustain EBI	• No dedicated role or resources to support a clinical champion (B)	*The people involved; Resources*
Research Co-production	• Collaborative partnership between researchers and health system to support ongoing sustainability	• Established partnership between research team and health system strategic clinical network (F) • Lack of clarity on the shift from research project to everyday practice (B)	*People involved; organizational setting*
***Identified in Key Informant Interviews***			
Remind clinicians	• Large posters on the unit and in the staff lounge	• Constantly visible and large in various areas of clinical practice and (F)	*Organizational Setting*
Audit and provide feedback	• Site visits accompanied by comprehensive written audit reports and brief summary ‘report cards’ based on observational feedback.	• Lack of buy-in regarding the clinical relevance of the EBI outcomes (B)	*Negotiating initiative processes*
Promote adaptability	• Tailored communication whiteboards • Volunteers services were adapted to support ongoing use	• Adaptation of implementation and sustainability is left to individual sites which helps to ensure alignment with local context (F) • Lack of clarity of implementation strategies can be adapted (B)	*EBI Design and Delivery*
Change record systems	• Alberta FICare is being integrated into an electronic clinical information system • Resources are posted to Alberta FICare websites	• Integration into electronic clinical information system supports use in practice (F) • Challenges with integration into workflow when other large-scale implementation projects are occurring simultaneously (B)	*Organizational setting*
Involve patients/consumers and family members	• Collected in the form of parent surveys, and real-time feedback, involvement in planning committees, and co-designing scale and spread resources	• Lack of time and opportunity to engage parents in providing feedback (B)	*People involved; Resources; The external environment*
Access new funding	• Resources needed for ongoing training and education • Secured 3 years of fixed funding to hire a provincial practice lead to coordinate and continue to evaluate and a peer family mentor clinical coordinator to further develop parent support	• Foundations commonly support family-centered initiatives (F) • Funding is not always available to support sustainability efforts (B)	*Resources*

##### Conduct ongoing training

Training was delivered in eLearning modules to be completed by multidisciplinary NICU staff. Since implementation, training has continued in various ways and intervals among different sites. The number of training and educational modules varied depending on whether it was targeted towards clinical champions, end users (all other multi-disciplinary staff), or unit clerks. Alberta FICare™ education has been largely integrated into new staff training at many sites. Following orientations, some sites include Alberta FICare™ in their annual orientation, while others complete “*education blitz’s*” (Participant 03). Education strategies also occurs in the form of regular emails.

##### Remind clinicians

Many participants described the use of posters to facilitate the sustainability of Alberta FICare™, as posters helped to remind clinicians about the intervention and provided resources for staff and parents. For example, at one site, two posters were located in the staff lounge, prompting major principles of Alberta FICare™ and outlining frequently asked questions.

##### Audit and provide feedback

Formal audit and feedback appeared to vary by site. During scale and spread efforts, formal audit and feedback was conducted quarterly. Audit and feedback were mentioned in the form of site visits (from 1 to 2 members of the Alberta FICare™ Project Team) accompanied by comprehensive written audit reports and brief summary ‘report cards’ based on observational feedback. The audit ‘report cards’ used a green, yellow, and red classification system to describe how the site was doing with use of the EBI components.

##### Change record systems

With the introduction of Connect Care (an electronic clinical information system) overlapping with the implementation of Alberta FICare™ at most sites, integrating the Parent Education component of Alberta FICare™ into their charting system played a significant role in supporting the sustainability of the new model of care. The integration work with Connect Care is ongoing and specific to the Parent Education component of Alberta FICare™.

##### Identify and prepare local champions

Participants identified strong clinical champions supported ongoing sustainability of Alberta FICare™. Clinical champions varied in their roles, but included nurse practitioners, clinical project managers, clinical nurse educators, and neonatologists.

##### Promote adaptability

Adapting the implementation and sustainability approach for Alberta FICare™ was a strategy used during scale and spread to make it easier for sites to implement and sustain the Alberta FICare™ practices. For example, some sites made changes to the layout of the communication whiteboards in patient rooms. Further, at one site, the addition of volunteer services to support the peer support program decreased the impact of Alberta FICare™ on staff workload.

##### Access new funding

Most participants identified that little resources were required to sustain Alberta FICare™. Some participants put an emphasis on the need for continued financial support to maintain sustainability into the future, including additional resources for staff training.

##### Involve patients/consumers and family members

Parent feedback was used as a strategy to facilitate sustainability by guiding how Alberta FICare™ should be implemented in the clinical setting. Throughout the scale and spread, parent feedback was mostly collected in the form of parent surveys and real-time feedback during fidelity audit site visits. Further, parents were involved in all planning meetings and co-designed spread and scale resources, including training modules.

#### Barriers and facilitators to using KT strategies for sustainability

In addition to the eight KT strategies identified in the environmental scan and key informant interviews, we also identified a range of barriers and facilitators to using the identified KT strategies for sustainability. These barriers and facilitators were categorized in all six constructs of the Consolidated Framework for Sustainability [35], including the people involved (*n* = 3), organizational setting (*n* = 3), resources (*n* = 3), negotiating initiative processes (*n* = 2), the external environment (*n* = 1), and EBI design and delivery (*n* = 1).

##### Conduct ongoing training

While the ongoing training and education was seen as helpful for orienting new staff, participants described barriers to completing the Alberta FICare™ modules. Some participants believed the educational strategies increased staff workload, despite funding available to backfill time for nurses to complete the learning modules. Further, there were some barriers with finding the educational materials which impacted its use, despite the materials being available via the internal website. One participant noted that accessibility is a barrier, explaining that “*if people have to go searching for something, it’s less likely to be used, right?*” (Participant 05).

##### Remind clinicians

The placement and size of the staff posters, and thus overall visibility, was reported to facilitate their impact as a reminder on Alberta FICare™. When asked how the posters facilitate the sustainability of Alberta FICare™, one participant explained “*they are at least a constant visual reminder to both parents and staff that this is a—I don’t want to say an expectation, but this is something that’s important to our unit. Just having that constant reminder that it’s there will definitely help with sustainability for sure*” (Participant 03).

##### Audit and provide feedback

Participants described challenges with the type of data collected in audit and feedback activities. The Alberta FICare™ dashboard reported on length of stay, ED visits, and readmissions. Participants described these data as being more useful for administrators and operational leaders than for point-of-care staff. As one participant noted, “*there’s so much that contributes to length of stay as well. You can’t just contribute it to Alberta FICare™*” (Participant 02).

##### Change record systems

Integrating Alberta FICare™ into the existing workflow proved to be a barrier, given many sites were implementing this alongside the implementation of an electronic clinical information system (Connect Care). However, there is ongoing work to integrate Alberta FICare™ into the electronic clinical information system, which participants described this as a key facilitator to supporting their workflow, saying “*having a Connect Care line with Alberta FICare™ promotes that Alberta FICare™ to continue and that nurses need to document on it. So that work has been really crucial, I think, in part of building sustainability*” (Participant 02). One example of integration is education points for ‘Parent Participation in Care’ and Bedside Rounds. This allows for providers to document parent integration in their infant’s care and bedside rounds.

##### Identify and prepare local champions

Participants noted that although clinical champions are a useful KT strategy for sustainability, it can be difficult to engage clinical champions when it is not a dedicated role, with explicitly dedicated resources. As one participant explained “*To make this sustainable you need resources dedicated to it. And to rely on frontline champions…it is difficult at the best times to get them engaged*” (Participant 02).

##### Promote adaptability

A significant barrier to adapting components of Alberta FICare™ to fit in the workplace has been the ambiguity of intervention tools and components. Alberta FICare™ was described as a model of care, and participants found it challenging to know what specific components should be adapted to sites to support sustainability. Some participants felt that it should not be up to the individual sites to develop tools to sustain the EBI, and in fact, there should be a more consistent approach to sustainability.

##### Access new funding

Participants noted that sustaining Alberta FICare™ has minimal financial requirements; however, funds are needed to support the use of KT strategies for sustainability. One participant explained their health centre foundation is a good resource for funding as they are “*good at supporting family initiatives and things that improve the family and patient experience*” (Participant 02).

##### Involve patients/consumers and family members

The peer family member support program was identified as a key intervention component that also supports the sustainability of Alberta FICare™; however, participants described challenges with engaging parents in the ongoing sustainability of Alberta FICare™. There was a lack of time and opportunity to engage parents in providing feedback. As one participant explained, “*parents are just in a state of crisis when they’re in the NICU’s. The last thing that they want to do is actually fill out a survey*” (Participant 04).

### Phase 3: alberta FICare™ lead consultation

Through correspondence with the Alberta FICare™ Leads, we learned of additional methods to enhance the KT strategies used for sustainability. For example, as part of the audit and feedback strategy, the Alberta FICare™ Leads developed an Alberta FICare™ dashboard. The Alberta FICare™ Leads provided additional details on the specific data reported in the dashboard, including length of stay, 7-day readmissions, and 7-day ED visits by site and zone. In relation to the peer family mentor support component of the EBI, COVID-19 delayed full implementation. Upon conclusion of scale and spread research efforts, the MNCY SCN began to assume leadership to support long-term sustainability of Alberta FICare™. There has been the development of a new MNCY role of provincial Family Mentor Clinical Coordinator aimed to complete implementation and support ongoing practice of the Family Mentor component. To accompany the educational strategies, they developed parent- and staff-facing webpages to communicate key details regarding Alberta FICare™ and support ongoing education on the initiative. Lastly, since the environmental scan and key informant interviews were conducted, the Alberta FICare™ team developed a business case to demonstrate the cost benefit of Alberta FICare™ and have since secured 3 years of fixed funding and hired a provincial Practice Lead to coordinate and continue to evaluate, and a Family Mentor Clinical Coordinator to further develop parent support. The Alberta FICare™ Leads presented before two provincial health services committees and secured funding based on the value generated by Alberta FICare™ for the health system and families.

## Discussion

This study aimed to examine *what* and *how* KT strategies are used to facilitate the sustainability of Alberta FICare™, an EBI that enhances family-centered care practice and empowers parents of infants admitted to the NICU with knowledge, skills, and confidence to facilitate an earlier discharge home [21, 22]. We conducted an environmental scan of relevant documents and key informant interviews with nursing clinical leaders and administrators to identify KT strategies used to sustain Alberta FICare™ and their perceived barriers and facilitators to using the KT strategies. By integrating the two data sources and seeking clarification and insights from Alberta FICare™ Project Leads, our findings provide a more comprehensive overview of *how* KT strategies are used for sustainability of EBIs. The environmental scan highlighted key KT strategies that were planned from the outset, including online education and clinical nurse champions. The key informant interviews identified additional KT strategies that were used at different sites, although not initially planned from the outset of the project (i.e., integrating components of Alberta FICare™ into the new electronic clinical information system, promoting adaptability). These insights demonstrated how KT strategies were selected and adapted over the sustainability process once an EBI is implemented into real-world practice and integrated into workflow processes. Our findings provide valuable information to support nurses and nurse leaders when selecting KT strategies to implement and sustain EBIs in a variety of clinical settings.

Both the environmental scan and key informant interviews highlighted training and educational strategies as one of the primary KT strategies for supporting sustainability of Alberta FICare™. Environmental scan documents described the use of online, asynchronous education modules for multidisciplinary NICU staff to support the ongoing delivery of Alberta FICare™. Similarly, the key informant interviews described staff education delivered via online learning modules, largely integrated into orientation training for new staff at several sites. The emphasis on educational strategies is not surprising. Our previous systematic review of KT strategies for implementing nursing guidelines identified 36/41 studies that used educational strategies, reporting positive impact on professional practice outcomes, professional knowledge outcomes, patient health status, and resource use outcomes [38]. Further, our scoping review of KT strategies used for the sustainability of EBIs (including models of care) found 24/25 studies reporting using educational strategies [20]. Despite educational strategies being the most commonly reported KT strategies, previous research clearly highlights the range of contextual factors influencing sustainability of EBIs, including inadequate staff resourcing and lack of organizational support [35, 39], which cannot be addressed by educational strategies alone [40].

The reported KT strategies were not employed in the same way across all sites represented in this study. For instance, the key informant interviews provided additional details on how educational strategies have been tailored to context-specific barriers and facilitators. Some sites have modified this KT strategy, including integrating educational strategies on Alberta FICare™ into their annual orientation, while others disseminate information in the form of regular emails. While it is important to avoid adaptations to the core EBI components, adapting and tailoring KT strategies to local barriers and facilitators is critical to support ongoing sustainability efforts [41].

Participants described an ad hoc approach to adaptations of KT strategies that lacked formal guidance. Our findings illustrate the need for clear guidance on *if* and *how* KT strategies used for initial implementation can be adapted for use in sustainability. This finding is consistent with previous sustainability studies. Johnson et al. 2019 conducted a qualitative content analysis of implementation studies funded by the United States National Institutes of Health and found that adaptation was not substantively described in their grant proposals [42]. Further, our scoping review identified a lack of reporting on how KT strategies are adapted from implementation to sustainability [20]. The lack of clarity on implementation to sustainability makes it challenging for nursing leaders to select, tailor, and use KT strategies for different types of EBIs. To address this gap, improved reporting efforts are needed to describe how KT strategies have been adapted to the local context, which will help to inform nurse leaders to select and tailor KT strategies to support the sustainability of EBIs. Implementation scientists have developed the Framework for Reporting Adaptations and Modifications to EBIs-Implementation Strategies (FRAME-IS), a practical tool for documenting and considering modifications to implementation strategies [43]. Our findings clearly indicate the need to use this type of reporting tool to expand our understanding of how to adapt implementation strategies into sustainability strategies.

This study demonstrated the value in the research co-production approach used by researchers and the health system [44]. This partnership was critical for the successful design, implementation, evaluation, and spread and scale of Alberta FICare™ across 14 NICUs in Alberta. However, some participants described Alberta FICare™ as primarily a research project, instead of a healthcare practice and policy change. In the environmental scan and key informant interviews, it was unclear who was primarily responsible for the ongoing maintenance of the EBI. Through the Alberta FICare™ Project Lead consultations, we learned that Alberta FICare™ now has three years of fixed funding, with a provincial Practice Lead to coordinate and continue to evaluate, and a Family Mentor Clinical Coordinator to further develop parent support.

A key strength of Alberta FICare™ is having ongoing, secure funding to support maintenance and ongoing use in practice. However, it is not always clear who is responsible for EBI sustainability in the co-production and sustainability literature. There is a lack of guidance to support researchers and health system leaders to engage in co-production past a research study or when grant funding ends [42]. Our study highlights several important practical questions for sustainability planning. What role do researchers have in sustainability of EBIs? Is there a distinct handover that has to occur or how does the health system ‘take over’ responsibility once an EBI has been deemed effective and successfully implemented? Other scholars highlight related considerations for sustainability work. Johnson et al.’s study of how researchers conceptualized and planned for the sustainability of health interventions, raised a similar question of who is responsible for sustainability planning, they recommend sustainability planning to be a “dynamic, multifaceted approach with the involvement of all those who have a stake in sustainability such as funders, researchers, practitioners, and program beneficiaries” [42]. The Alberta FICare™ Project Leads highlight the value in this dynamic, multifaceted approach that allowed them to work with their funders to secure resources to support sustainability. Further, these findings speak to the need for longitudinal research on the sustainability process. Sustainability of EBIs is more than a single snapshot in time, and ongoing evaluation is needed to understand how it works in practice with research co-production partnerships between researchers, health system leaders, and patients and families.

The science on KT strategies is evolving. For this study, we used the 2015 version of the ERIC Taxonomy to guide our data collection and analysis activities [7]. Since then, an important sustainability science paper has been published where researchers adapted, refined, and extended the ERIC compilation to incorporate an explicit focus on sustainment [45]. Nathan et al. [45] found that most ERIC strategies required minor changes, whereas four strategies were significantly revised. Most notably, “develop educational materials” was adapted to “review and update educational materials” which aligns with our findings on the need for ongoing updates to educational materials for Alberta FI-Care™. Overall, our study complements Nathan et al.’s sustainment-explicit ERIC glossary by describing *how* these strategies support sustainability with practical and illustrative examples from Alberta FI-Care™. Moving forward, efforts are needed to apply this sustainment-explicit ERIC glossary to other EBI projects to further develop our understanding of *what* and *how* KT strategies are being used to implement and sustain EBIs.

We identified two conceptual challenges that require further exploration in the implementation and sustainability science literature. First, a challenge with examining sustainability of an EBI is navigating the difference between EBI implementation and sustainability. This study supports the need to shift our perspective of implementation and sustainability to a continuum instead of distinct entities [46]. Lennox et al.’s systematic review on sustainability approaches in healthcare revealed two distinct conceptualizations of sustainability: (i) Sustainability is a linear process that follows implementation, it is the end goal to be achieved; and (ii) Sustainability is a concurrent process alongside implementation, where the process is to be influenced and adapted over time to impact long-term use of the intervention [35]. Our study findings highlight the value in a concurrent approach. While Alberta FICare™ was successfully implemented, it is unclear when or how an implementation strategy became a sustainability strategy. Building on the reporting guideline work for implementation researchers, we recommend that researchers also adequately report KT strategies for sustainability, as well as adaptation of KT strategies from implementation to sustainability to support replication by other researchers, clinicians, and implementation practitioners. Such details include KT strategy dose, frequency, mode of delivery, and adaptations from initial implementation efforts to long-term sustainability efforts.

Second, Moore et al. 2017 cite two foundational challenges with the sustainability literature: (i) lack of standard definition and (ii) variety of synonyms used in the literature. Our study findings highlight an additional challenge with terminology; sustainability often gets combined with spread and scale, despite distinct differences [30]. Greenhalgh and Papoutsi define spread as “replicating an initiative somewhere else” and scale as “building infrastructure to support full scale implementation” [18]. However, sustainability differs from these two processes and focuses more on the extent to which an EBI can deliver its intended benefits over an extended period of time after external support is terminated [47]. In our environmental scan, documents primarily described the process for moving from the cRCT towards scale and spread of the EBI into all NICUs in the province. This was a critical process to successfully increase the use of Alberta FICare™ across more healthcare institutions. However, documentation lacked detailed information about KT strategies to facilitate *sustainability* of the EBI once the EBI had been scaled and spread. Similarly, our key informant interviews reiterated the success of scale and spread but described a lack of clarity of what KT strategies to use to support sustainability over time. Future EBI scale and spread initiatives should also consider sustainability planning from the outset. Further, additional research is needed to understand if sustainability strategies change based on if the focus of the EBI is on spread or on scale.

### Nursing implications

There are specific implications from our study for nursing practice and research. We echo Proctor et al.’s calls for a more intentional sustainability research agenda, including advancing the capacity, culture, and mechanisms for sustainability and advancing methods for sustainability research [16]. Advancing this agenda within the nursing context is critical given the significant role nurses play in the implementation and sustainability of EBIs in healthcare [48, 49]. Implementation capacity building is becoming increasingly common given the importance of assessing barriers and facilitators to practice change to inform implementation planning [50]. However, often these initiatives focus on individual provider behaviors and context of the EBI implementation. Nursing clinicians need tangible tools to support their sustainability planning as well. Capacity building efforts are needed to support nursing practitioners, leaders, and health system administrators to tackle EBI implementation and sustainability on a continuum and start to plan for sustainability from the start of a nursing practice or policy change initiative.

As nursing researchers, it is our role to advance the science of implementation and sustainability and support nurses and administrators to use evidence-based KT strategies in their implementation and sustainability efforts. To do so, further research is needed to build on the implementation science body of knowledge and think about sustainability-specific strategies or how to adapt implementation strategies to be sustainability strategies and support the maintenance of EBIs in nursing practice and policy. We recommend building on existing sustainability frameworks, such as the CFS and the Dynamic Sustainability Framework, to support reporting and testing initiatives of KT strategies for sustainability. Lastly, nursing researchers must work in a research co-production approach to successfully enable sustainability. As our findings indicate, the research partnerships between University of Calgary and the AHS MNCY SCN allowed for rigorous research, scale and spread, and the establishment of secured funding to support ongoing sustainability. The cRCT and process evaluation approach of the Alberta FICare™ provided the evidence to scale and spread the EBI across the province. These were critical steps in advancing the sustainability of the EBI. Oftentimes, sustainability is thought about retrospectively: An EBI is implemented, and now we want to sustain it. We urge researchers, nursing leaders, and health system administrators to work together in prospective sustainability research and pragmatic planning.

### Strengths and limitations

Our study findings should be considered with the following limitations in mind. This study was conducted in partnership with health system knowledge users; however, we did not have patient and public involvement in our study. Having patient and public partners on this study would add insights into the relevancy and utility of the KT strategies identified. The study sample for the qualitative interview phase may have missed some important perspectives. We did not interview a key informant from each NICU that implemented Alberta FICare™. As such, we may have missed KT strategies that are being used to facilitate sustainability in different contexts. Further, we did not interview point of care nurses to explore how they are using the EBI in their daily practice. Despite these limitations, we supplemented interviews with the environmental scan document analysis and Alberta FICare™ Project Leads consultation, which allowed for a broader understanding of *what* and *how* KT strategies are used to facilitate the sustainability of Alberta FICare™. Further, we used several implementation and sustainability frameworks to map findings onto existing literature on KT strategies.

## Conclusion

This multimethod qualitative study explored how KT strategies are used to facilitate the sustainability of an EBI. Using Alberta FICare™ as a case example, we identified a range of KT strategies used for sustainability, including online education, clinical nurse champions, and academic-health system co-production. Our findings illustrate how KT strategies are adapted over the sustainability process once an EBI is implemented into real-world nursing practice. Adaptation of interventions must be considered from the onset of implementation so interventions can be tailored to align with contextual barriers for sustainability. Further, this research highlights the importance of clearly articulating who is responsible for continued championing for the sustainability of EBIs. Clear guidance is needed to continually support researchers and nurse leaders in co-producing strategies that facilitate the long-term sustainability of effective EBIs in nursing practice and policy.

### Electronic supplementary material

Below is the link to the electronic supplementary material.

Supplementary Material 1

## Data Availability

All data generated or analysed during this study are included in this published article.

## Abbreviations

AHS: Alberta Health Services
AIMD: Aims, ingredients, mechanism, delivery
CFS: Consolidated framework for sustainability
cRCT: Clustered randomized control trial
EBI: Evidence-based interventions
ED: Emergency Department
ERIC: Expert Recommendations for Implementing Change
FICare: Family Integrated Care
FRAME-IS: Framework for Reporting Adaptations and Modifications to EBIs-Implementation Strategies
KT: Knowledge Translation
LOS: Length of stay
MNCY: Maternal, Newborn, Child & Youth
NICU: Neonatal Intensive Care Unit
SCN: Strategic Clinical Network
