# Supplementary material for: Knowledge translation strategies used for sustainability of an evidence-based intervention in child health: a multimethod qualitative study
Source: BMC Nurs. 2024 Feb 17;23:125. doi: 10.1186/s12912-024-01777-4 (PMC10874067; doi:10.1186/s12912-024-01777-4)
Supplement: Supplementary file 1 — Supplementary Material 1 [file 12912_2024_1777_MOESM1_ESM.pdf]

Appendix 1. Interview Guide

Interview topic guide for FiCare sustainability KT strategies

Interview Guide

1. Can you describe FiCare? For instance, can you tell me the core components of the intervention, when and why it was implemented?

2. Can you describe some of the things that were (are being) done to ensure that FICare will be sustained in the future?

   Prompts:

   a) For instance, can you tell me whether the same KT strategies were used for implementation and sustainability?
   b) Are these strategies still being used?

      Probes: Use of strategies to:

      a. Develop stakeholder interrelationships;
      b. Train and educate stakeholders;
      c. Support clinicians;
      d. Engage patients and families;
      e. Utilize financial strategies;
      f. Change infrastructure.
      g. Use of evaluative and iterative strategies;
      h. Provide interactive assistance;
      i. Adapt and tailor to context

3. How have you been involved in *the implementation and /or sustainability of FiCare*?

   Prompts:

   a) What is your role?
   b) How do you feel about your level of involvement?

c) What are your main reasons for supporting the continuation of FiCare or not?
d) Does *the intervention* make sense to you? (how, why)

4. How was *FiCare* designed and delivered (CFS 1)?

   Prompts:

   a) Evidence-base for FiCare?
   b) Training and capacity building strategies used?
   c) Improvement methods used?
   d) How has progress been monitored and feedback provided over time?
   e) How have the design and delivery strategies used influenced sustainability of *FiCare*?

5. Who is or was involved in the sustainability of the FiCare (CFS 3)?

   Prompts:

   a) How has your involvement influenced your perceptions of sustainability?
   b) From your perspective, who is driving the sustainability of *FiCare* in your work environment/ organization?
   c) To what extent are sustainability efforts collaborative in nature? Stakeholder involvement (staff, patients).
   d) Are there any leaders or champions that have facilitated or hindered the sustainability of FiCare? In what ways?
   e) Can you tell me about any relationships or collaborative networks that have been important for the sustainability of FiCare? How have these networks influenced sustainability?

6. In what ways is FiCare being sustained or not (CFS 2)?

   Prompts:

   a) To what extent do you believe *FiCare* has become part of 'daily work' in your work/organization?
   b) From your perspective, who is driving the sustainability of *FiCare* in your work environment/ organization?

c) How has the continuation of FiCare influenced your workload?
d) How simple or difficult have you found FiCare to sustain, why?
e) How does FiCare align with the aims of your unit or organization?

7. Are there any resources that have helped or prevented the sustainability of FiCare and how (CFS 4)?

   Prompts:

   a) Staff resources that have helped or prevented the sustainability of FiCare and how?
   b) Time resources that have helped or prevented the sustainability of FiCare and how?
   c) Infrastructure resources that have helped or prevented the sustainability of the FiCare and how?
   d) Other general resources that have helped or prevented the sustainability of FiCare and how?
   e) Funding resources that have helped or prevented the sustainability of FiCare and how?

8. How has FiCare been adapted or modified in response to new evidence or contextual influences?

   Prompt:

   Have any components of FiCare continued? If so, which components and how?

   What components remain the same?

9. Can you tell me about the organizational setting (i.e. your unit and/or AHS) and how it has influenced the sustainability of FiCare(CFS 5)?

   Prompts:

   a) In what ways has organizational readiness and capacity influenced sustainability of FiCare?
   b) How does FiCare align with your organization's values and culture?
   c) Has FiCare been integrated with existing programs and policies? how?

   d) Have new policies/procedures from FiCare been continued in practice?

10. How has the external environment facilitated or hindered sustainability of FiCare(CFS 6)?

    Prompts:

    a) Any Socioeconomic and political considerations?
    b) Has awareness and attention of the clinical issue and FiCare been continued? How?
    c) Has there been spread of FiCare to other organisations? Describe?

11. Do you feel that FiCare has had any benefits for patients and staff? What are these benefits and how have they continued or not?
